# Child feeding practices and diarrheal disease among children less than two years of age of the nomadic people in Hadaleala District, Afar Region, Northeast Ethiopia

**DOI:** 10.1186/s13006-017-0115-z

**Published:** 2017-06-05

**Authors:** Zemichael Gizaw, Wondwoson Woldu, Bikes Destaw Bitew

**Affiliations:** 1 Department of Environmental and Occupational Health and Safety, Institute of Public Health, College of Medicine and Health Sciences, University of Gondar, Gondar, Ethiopia; 2 Hadaleala District Health Office, Hadaleala District, Afar Regional State Ethiopia

**Keywords:** Childhood diarrhea, Less than two years of age, Child feeding practices, Nomads, Afar Region

## Abstract

**Background:**

Diarrhea is a serious public health problem in Ethiopia. It is responsible for 24–30% of all infant deaths and there is a lack of evidence on the health burdens among the nomadic people. This study was therefore designed to assess the prevalence of diarrhea among children less thanvtwo year’s of age and its association with feeding practices among the nomadic people in Hadaleala district, northeast Ethiopia.

**Methods:**

A cross-sectional study was conducted in Hadaleala district. A total of 367 children less than two years of age were included using the multistage cluster sampling technique. Data were collected by a structured questionnaire. Multivariable binary logistic regression analysis was used to identify variables associated with diarrheal disease.

**Results:**

The prevalence of diarrhea among children less than two year’s of age during the two week period was 31.3% (95% CI, 25.9, 36.1%). Diarrhea occurrence was associated with; children aged between 6–11 months (AOR 6.28, 95% CI, 3.00, 13.12), aged between 12–24 months (AOR 6.21, 95% CI, 3.13, 12.30), illiterate mothers (AOR 6.61, 95% CI, 2.27, 19.21), delay to initiate early breastfeeding for children aged less than six months (AOR 9.13, 95% CI, 1.78, 46.72), children less than six months of age not currently exclusively breastfed (AOR 13.33, 95% CI, 1.59, 112.12), delay to initiate early breastfeeding for children aged 6–24 months (AOR 2.87, 95% CI, 1.49, 5.51), no breastfeeding at the time of the survey (AOR 3.51, 95% CI, 1.57, 7.82), children aged 6–24 months who didn’t exclusively breastfeed in the first six months (AOR 19.24, 95% CI, 8.26, 44.82), consuming uncooked foods (AOR 6.99, 95% CI, 2.89, 16.92), not eating cooked foods immediately after cooking (AOR 3.74, 95% CI, 1.48, 9.45), hand washing with only water (AOR 24.94, 95% CI, 6.68, 93.12), and rotavirus vaccination (AOR 0.09, 95% CI, 0.03, 0.29).

**Conclusions:**

The prevalence of diarrhea among children less than two year’s of age in Hadaleala district was high. To prevent diarrhea, the mothers should start breastfeeding early and practice exclusive breastfeeding. Moreover, mothers should improve the hygiene of supplementary foods.

## Background

Diarrhea is a leading cause of child deaths, accounting for 9% of all deaths among children less than five years of age worldwide in 2015. This translates into over 1,400 young children dying each day, or about 530,000 children a year. Most deaths from diarrhea occur among children less than two years of age in South Asia and sub-Saharan Africa [1–3].

Diarrheal disease is a serious public health problem among children in Ethiopia. In Ethiopia, during 2011, about 13% of children less than five years of age had diarrhea [4]. In the country, 24–30% of all infant deaths were due to diarrhea [5]. A multiregional baseline household health status survey indicated that the two week prevalence of any diarrhea among children aged 0–23 months was reported to be 22% [6]. As a part of the country, Afar region is one of the poorest, least developed and under-serviced regions of Ethiopia where the highest child mortality rate is reported. Rural communities of the region are suffering from shortage of water, hygiene and sanitation facilities. The main sources of water for the community are rivers, streams, ponds, and wells. During 2015, safe water and sanitation coverage of the district was 35% and 12%, respectively [7].

Childhood diarrheal disease among less than two year aged children is a result of the interactions of many factors; socioeconomic, environmental, behavioral factors, and child feeding practices. Literature show that child feeding practices had a direct link with childhood diarrhea. Early breastfeeding initiation, maintenance of breastfeeding, complementary feeding, time to the start of complementary feeding, hygiene of complementary foods, and child vaccination were some of the practices associated with childhood diarrhea. Colostrum or breast milk, which is rich in nutrients, has benefits to minimize infectious diseases, primarily acute diarrhea [8, 9]. Early initiation and maintenance of breastfeeding is the most appropriate form of nourishment for ensuring adequate growth and immunologic development of children. Breast milk provides all essential nutrients that are important for brain, nervous system, intellectual, neurological, psychomotor, and social development of children. It reduces the risk of infections and their associated mortality and morbidity [10–17]. Childhood diarrhea is a serious public health challenge among children who started complementary feeding. The hygiene of complementary foods is a risk factor associated with complementary feeding and unhygienically prepared and handled complementary foods may contain disease causing pathogenic microorganisms [18–25].

The health burdens of diarrheal disease among children less than two years of age are widely recognized at global level. Despite the fact that, its prevalence and the association with feeding practices among the nomadic people of Ethiopia are not well researched, and there is a lack of evidence among the nomadic people. This community based cross-sectional study was therefore designed to assess the prevalence of diarrheal disease in children aged less than two years and its association with child feeding practices among nomadic people in Hadaleala district, Afar Region, northeast Ethiopia. The results of this study will help the community to design and implement strategies to prevent or minimize childhood diarrheal disease. Furthermore, it may also fill the literature gaps and may act as a baseline data for further studies.

## Methods

### Study design and settings

A community based cross-sectional study was conducted in Hadaleala district, Afar Region, northeast Ethiopia in May, 2015. Hadaleala district is one of the districts of Hariresu zone, Afar Regional State. It is located at 341 km southwest of the regional capital, Semera, and 268 km north of Addis Ababa, the capital city of Ethiopia. It has an area of 1272 km^2^ divided into 11 rural kebeles (the smallest administrative units in Ethiopia) with a total population of 42,845 as projected for the year 2015. It has 7,516 households with an average household size of 5.7 persons per house. The number of children aged less than five years account for 10.1% (4,328) of the total population. The population is very scattered, and the average population density is 14 persons/km^2^. The economy of the district is based on livestock and crop production [7] and due to the dispersed pasture and water resources, the communities in the district are mobile or nomadic.

### Sampling size determination and procedure

The multistage cluster sampling technique was used to select study participants from the nomadic population. The clusters were villages with defined geographical boundaries. Out of a total of 11 kebeles, six were selected by the simple random sampling technique. The six selected kebeles were clustered into 39 villages, and 17 were selected by the systematic random sampling technique. The sampled kebeles and villages were nearly 55% and 44%, respectively, which is representative to the source population. Hence, the sampling procedure is multistage cluster sampling, all households (367) with children less than two years of age were included in the study.

### Measurement of outcome variable

Diarrheal disease among children less than two years of age, the primary outcome variable of this study, is defined as having three or more loose or watery stools in 24 hours [26, 27]. The prevalence of childhood diarrheal disease within the two week period prior to data collection was calculated as the total number of diarrhea cases divided by 367 (the total number of children aged less than two years of age who were participating in the study).

### Data collection tools and procedures

A pretested structured questionnaire was used to collect data. The questionnaire was prepared in English and translated to the local language and back translated to English to maintain the consistency of the questions. The tool was pre-tested out of the study area in a community which had similar characteristics prior to the actual data collection. To improve the quality of the data, eight diploma graduate nurses and two environmental health officers who were fluent enough, both in Amharic and Afarigna (local languages) and working in the district were involved in the data collection process. After the pretest and training, the data collectors visited all households in the selected clusters. The youngest (at the time of the survey) children were included in the study when there were more than one child aged less than two years of age in the household. Finally, the collected data were checked and corrected by the data collectors immediately after finalizing the questionnaire. Supervisors daily checked the completeness, quality, and consistency of information collected.

### Data management and statistical analysis

Data were entered using the Epi-Info version 3.5.3 statistical package and exported to SPSS version 20 for further analysis. For most variables, data were presented by frequencies and percentages. The univariable binary logistic regression analysis was used to choose variables for the multivariable binary logistic regression analysis and variables which had less than 0.2 *p* – values by the univariable analysis were then separately analyzed by the multivariable binary logistic regression for controlling the possible effects of confounders. Variables which have significant associations were identified on the basis of the adjusted odds ratio (AOR) with a 95% confidence interval and *p* < 0.05. Normality and Hosmer and Lemshow test were done to assure the normal distribution of continuous variables and to check out the model fitness, respectively.

## Results

### Socioeconomic characteristics of respondents

A total of 367 mothers-children pairs had participated in the study with a 100% response rate. The median age of the children was 11 months, and the interquartile range (IQR) was 5–17 months. Only 14 (3.8%) of the households had three children. One hundred ninety-four (52.9%) of the children were male, and 158 (43.1%) of the children were aged between 12–24 months. Nearly half, 182 (49.6%) of the mothers were aged between 25–34 years. The mean age of the mothers was 27.6 years (with ± 6.0 standard deviation). Almost all, 360 (98.1%) of the mothers were currently married. The great majority, 321 (87.5%) of mothers had no formal education. Two hundred twenty (59.9%) of the households had more than five family members. About 258 (70.3%) households were economically poor (Table 1).

**Table 1:** Socioeconomic information of households with children less than –two years of age (*n* = 367) in Hadaleala District, Afar Region, Northeast Ethiopia, May, 2015

Variables	Frequency	Percent
Number of children less than two years of age in the house		
One	212	57.8
Two	141	38.4
Three	14	3.8
Age of children (months)		
< 6	113	30.8
6–11	96	26.2
12–24	158	43.1
Sex of children		
Male	194	52.9
Female	173	47.1
Age of mothers (years)		
15–24	126	34.3
25–34	182	49.6
≥ 35	59	16.1
Marital status of mothers		
Currently married	360	98.1
Not currently married	7	1.9
Educational status of mothers		
No formal education	321	87.5
Formal education	46	12.5
Family size		
≥ 5	220	59.9
< 5	147	40.1
Economic status of households		
Poor	258	70.3
Medium	109	29.7

### Child feeding practices

The status of breastfeeding of children less than two years of age was determined by this survey. All the children aged less than six months (*n* = 113) were fed breast milk at the time of the survey. However, 48 (42.5%) of the children did not feed breast milk exclusively, and also received complementary foods. One-third, 38 (33.6%), of the children didn’t receive breast milk within one hour immediately after birth.

Similarly, the breastfeeding status of children aged 6–24 months was determined. From a total of 254 children, 188 (74.0%) were fed breast milk exclusively for the first six months. On the other hand, only 47 (18.5%) of the children aged between 6–24 months were not being breastfed at the time of the survey. One hundred fifty-four (60.6%) children received breast milk within one hour immediately after birth.

About half, 131 (50.6%) of children aged less than two years, who started complementary feeding were given uncooked foods, like raw milk and nearly three–fourth, 191 (73.8%) of them have been given foods immediately after cooking. One hundred nineteen (32.4%) households who had children aged less than two years fetched drinking water from protected sources. More than half, 208 (56.7%) of the mothers have been washing their hands with water only before preparing child meal and feeding their children. Nearly one-third, 115 (31.3%) of the children received rotavirus vaccine (Table 2).

**Table 2:** Child feeding practices of households (*n* = 367) in Hadaleala District, Afar Region, Northeast Ethiopia, May, 2015

Variables	Frequency	Percent
Children who ate uncooked food (*n* = 259)	131	50.6
Children who ate cooked foods immediately (*n* = 259)	191	73.8
Children who received rotavirus vaccination (*n* = 367)	115	31.3
Mothers who washed their hands before food preparation and feeding the child with only water (*n* = 367)	208	56.7
Mothers who washed their hands before food preparation and feeding the child with soap (*n* = 367)	159	43.3
Drinking water sources (*n* = 367)		
Protected	119	32.4
Unprotected	248	67.6

### Prevalence of diarrheal disease among less than two years of age children

**Fig. 1:**
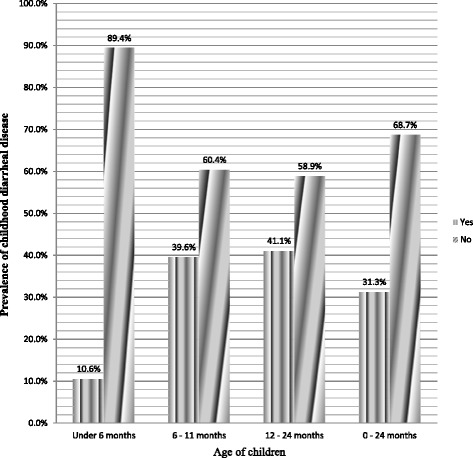
Prevalence of diarrhea disease in different age categories of children less than two years of age in Hadaleala district, Afar Region, Northeast Ethiopia, May 2015

A total of 115 children less than two years of age had diarrhea in the two week period prior to data collection. Therefore, the two week period prevalence of diarrhea among children less than two years of age was found to be 31.3% (95% CI, 25.9, 36.1%). In addition, 58 children had diarrhea at the time of data collection, therefore, the point prevalence was found to be 15.8% (95% CI, 12.3, 19.7%). Nearly half, 57 (49.6%) of the children who had diarrhea obtained treatment from public health facilities and 58 (50.4%) were treated at home. The age specific prevalence of diarrhea was highest among children aged 12–24 months, at 65 (41.1%) (Fig. 1).

### Factors associated with childhood diarrheal disease

Sociodemographic variables like age of children, sex of children, education status of mothers, marital status of mothers, family size, number of children less than two years of age, age of mothers, and household economic status were analyzed by Univariable binary logistic regression to identify the variables associated with childhood diarrheal disease among children less two years of age. Only a child’s age and mother’s education level had a *p* – value less than 0.20 and were further analyzed by the multivariable binary logistic regression model, and both of them were statistically associated with childhood diarrhea (Table 3). Children aged 6–11 months were 6.28 times more likely to be affected by diarrheal disease compared with children aged less than six months (AOR 6.28, 95% CI, 3.00, 13.12). Similarly, the likelihood of diarrheal disease was 6.21 times more likely to be higher among children aged between 12–24 months (AOR 6.21, 95% CI, 3.13, 12.30). The occurrence of diarrheal disease among children less than two years of age was 6.61 times higher when their mothers had no formal education compared with their counterparts (AOR 6.61, 95% CI, 2.27, 19.21).

**Table 3:** Sociodemographic variables associated with diarrheal disease among children less than two years age (*n* = 367) in Hadaleala District, Afar Region, Northeast Ethiopia, May, 2015

Socioeconomic variables	Diarrheal disease		Crude Odds Ratio (95% Confidence Interval)	Adjusted Odds Ratio (95% Confidence Interval)
	Yes	No		
Age of children (months)				
< 6	12 (10.6%)	101 (89.4%)	1	
6–11	38 (39.6%)	58 (60.4%)	5.51 (2.67, 11.39)	6.28 (3.00, 13.12)**
12–24	65 (41.1%)	93 (58.9%)	5.88 (2.99, 11.58)	6.21 (3.13, 12.30)**
Mothers’ education level				
No formal education	111 (34.6%)	210 (65.4%)	5.55 (1.94, 15.88)	6.61 (2.27, 19.21)*
Formal education	4 (8.7%)	42 (91.3%)	1	

_*_Statistically significant variables at *p* = 0.001 _**_Statistically significant variables at *p* < 0.001

The result of Hosmer and Lemshow test was > 0.857

Early initiation of breastfeeding and child feeding status (whether exclusively breastfed or not) were entered into the binary logistic regression model and found to be associated with diarrheal disease among children aged less than six months (Table 4). Children who didn’t start breastfeeding within one hour immediately after birth had a greater chance to develop diarrheal disease (AOR 9.13, 95% CI, 1.78, 46.72). The likelihood of diarrhea was 13.33 times higher among children who didn’t exclusively receive breast milk compared with children who received breast milk exclusively (AOR 13.33, 95% CI, 1.59, 112.12).

**Table 4:** The association between child feeding practices and diarrheal disease among children less than 6 months of age (*n* = 113) in Hadaleala District, Afar Region, Northeast Ethiopia, May, 2015

Breastfeeding variables	Diarrheal disease		Crude Odds Ratio (95% Confidence Interval)	Adjusted Odds Ratio (95% Confidence Interval)
	Yes	No		
Early breastfeeding initiation				
Yes	2 (2.7%)	73 (97.3%)	1	
No	10 (26.3%)	28 (73.7%)	13.04 (2.69, 63.25)	9.13 (1.78, 46.72)**
Breastfeeding status				
Not exclusive breastfeeding	11 (22.9%)	37 (77.1%)	19.03 (2.36,153.33)	13.33 (1.59, 112.12)*
Exclusive breastfeeding	1 (1.5%)	64 (98.5%)	1	

_*_Statistically significant variables at *p* < 0.05 **Statistically significant variables at *p* < 0.01

The result of Hosmer and Lemshow test was > 0.216

Similarly, initiation of early breastfeeding within one hour after birth, breastfeeding status at the time of the survey, and child feeding status in the first six months were also associated with diarrheal disease among children aged between 6–24 months (Table 5). Children aged between 6–24 months who didn’t start breastfeeding within one hour after birth were 2.87 times more likely to be affected by diarrheal disease compared with their counterparts (AOR 2.87, 95% CI, 1.49, 5.51). Also, when breastfeeding was not started immediately after birth, childhood diarrheal disease was 3.51 times higher among children who didn’t maintain breastfeeding at the time of the survey (AOR 3.51, 95% CI, 1.57, 7.82). Children aged between 6–24 months, who were not exclusively breastfed in the first six months, had a greater chance to have diarrhea (AOR 9.24, 95% CI, 8.26, 44.82).

**Table 5:** The association between breastfeeding practices and diarrheal disease among children aged between 6–24 months (*n* = 254) in Hadaleala District, Afar Region, Northeast Ethiopia, May, 2015

Breastfeeding variables	Diarrheal disease		Crude Odds Ratio (95% Confidence Interval)	Adjusted Odds Ratio (95% Confidence Interval)
	Yes	No		
Early breastfeeding initiation				
Yes	40 (26.0%)	114 (74.0%)	1	
No	63 (63.0%)	37 (37.0%)	4.85 (2.82, 8.35)	2.87 (1.49, 5.51)*
Breastfeeding at the time of the survey				
Yes	72 (34.8%)	135 (65.2%)	1	
No	31(66.0%)	16 (34.0%)	3.63 (1.86, 7.08)	3.51 (1.57, 7.82)*
Breastfeeding status in the first 6 months				
Not exclusive breastfeeding	58 (87.9%)	8 (12.1%)	23.04 (10.23, 51.87)	19.24 (8.26, 44.82)**
Exclusive breastfeeding	45 (23.9%)	143 (76.1%)	1	

*Statistically significant variables at *p* < 0.05 **Statistically significant variables at *p* < 0.01 | The result of Hosmer and Lemshow test was > 0.995

Finally, consumption of uncooked foods, eating foods immediately after cooking, hand washing before food preparation and feeding the child, child vaccination, and drinking water sources were analyzed by the Univariable binary logistic regression to choose for the multivariable binary logistic regression. Consumption of uncooked foods, eating foods immediately after cooking, hand washing practices, and child vaccination had a *p* – value less than 0.20 and then analyzed by the multivariable binary logistic regression and found to be associated with diarrheal disease among children less than two years of age (Table 6). Children who consumed uncooked foods were 6.99 times more likely to be affected by diarrheal disease (AOR 6.99, 95% CI, 2.89, 16.92). Childhood diarrheal disease among children who didn’t eat foods immediately after cooking was 3.74 times higher compared with their counterparts (AOR 3.74, 95% CI, 1.48, 9.45). Mother’s hand washing practice before food preparation and feeding the children was also associated with the occurrence of diarrhea. Childhood diarrhea was higher among children whose mothers washed their hands with water only before food preparation and feeding the children compared with mothers who washed with soap (AOR 24.94, 95% CI, 6.68, 93.12). Furthermore, childhood diarrhea was associated with rotavirus vaccination.Childhood diarrheal disease was reduced by 91% among children who received rotavirus vaccination (AOR 0.09, 95% CI, 0.03, 0.29).

**Table 6:** Correlates of childhood diarrheal disease among children less than 2 years of age in Hadaleala District, Afar Region, Northeast Ethiopia, May, 2015

Variables	Diarrheal disease		Crude Odds Ratio (95% Confidence Interval)	Adjusted Odds Ratio (95% Confidence Interval)
	Yes	No		
Hand washing practice (*n* = 367)				
With water only	112 (53.8%)	96 (46.2%)	60.67 (18.75, 196.35)	24.94 (6.68, 93.12)**
With soap	3 (1.9%)	156 (98.1%)	1	
Serving uncooked foods (*n* = 259)				
Yes	88 (67.2%)	43 (32.8%)	15.42 (8.05, 29.55)	6.99 (2.89, 16.92)**
No	15 (11.7%)	113 (88.3%)	1	
Feeding cooked foods immediately after cooking (*n* = 259)				
Yes	50 (26.2%)	141 (73.8%)	1	
No	53 (77.9%)	15 (22.1%)	9.96 (5.16, 19.24)	3.74 (1.48, 9.45)*
Child received rotavirus vaccination (*n* = 367)				
Yes	5 (4.3%)	110 (95.7%)	0.06 (0.02, 0.15)	0.09 (0.03, 0.29)**
No	110 (43.7%)	142 (56.3%)	1	
Drinking water source cp				
Protected	9 (7.6%)	110 (92.4%)	1	
Unprotected	106 (42.7%)	142 (57.3%)	9.12 (4.42, 18.83)	1.55 (0.47, 5.12)

*Statistically significant variables at *p* < 0.01, **Statistically significant variables at *p* < 0.001. The result of Hosmer and Lemshow test was > 0.974

## Discussion

This study investigated the prevalence of diarrheal disease and child feeding practices among children less than two years of age in Hadaleala district. The two week period prevalence of diarrheal disease was 31.3% (95% CI 25.9, 36.1%). The prevalence seen in this study is higher than the national prevalence, which was 23–25% [4]. The magnitude of childhood diarrheal disease reported by this study is also higher than the prevalence reported in three different regions of Ethiopia; Tigray (17%), Amhara (18%) and Oromiya (25%) [6] and studies done in northeastern Brazil (22.9%) [28] and Saudi Arabia (24%) [29]. The high incidence in the current study might be attributed to the difference in the sociodemographic, environmental, and behavioral characteristics of households and the nomadic nature of the population. Nomads migrate from place to place in search of pasture and water. Having no permanent residential places, they may not have access to basic health care, water and sanitation services. Their main sources of water are rivers, streams, and wells which are prone to contamination. The nomads practice open defecation and their living environment is polluted with human excreta, the main risk factor for diarrheal disease, especially for the children who routinely play in the unhygienic environment. Moreover, the people suffer from illiteracy and poverty which effects their quality of life. All these phenomena are the direct risk factors for the high prevalence of childhood diarrheal diseases [7].

In this study, it was found that diarrheal disease was associated with age of the children. The odds of having diarrheal disease were higher among children aged 6–11 and 12–24 months compared to children aged less than six months. This may be due to the fact that children aged more than six months start crawling or walking which increases their exposure to infectious agents. Furthermore, when children aged less than six months start complementary feeding this may increase their exposure to different types of infections through contaminated food and water [30–36].

This study identified that childhood diarrheal disease was statistically associated with the educational status of mothers. Children whose mothers had attended formal education (primary and above) were less likely to develop diarrhea compared to children whose mothers who had not attended any formal education. Receiving an education may increase awareness or knowledge about the transmission and prevention methods of diarrhea, enhanceing household health and sanitation practices, and encourages changes in behavior at the household level [37–42].

In this study, we found that diarrheal disease was associated with early initiation and maintenance of breastfeeding. Diarrhea was more common among children who didn’t start breastfeeding within one hour immediately after birth and among children who didn’t maintain breastfeeding at the time of the survey. Other studies also reported similar findings with this study [19, 28, 43]. This might be due to the fact that children who didn’t start breastfeeding within one hour after birth and didn’t maintain breastfeeding may fail to get the benefits of colostrum, which is rich by minerals and vitamins. Breast milk decreases both the incidence and severity of infectious diseases, primarily acute diarrhea [8, 9]. Early initiation and maintenance of breastfeeding is also the most appropriate form of nourishment for ensuring adequate growth and immunologic development of children [10–13]. Breastfeeding confers short term and long term benefits [15]. It helps to protect children against a variety of acute and chronic disorders [16, 17].

The childhood diarrheal disease was statistically associated with feeding status of children in the first six months of age. The current study explored that those children who didn’t receive breast milk exclusively in the first six months had a greater chance to develop diarrhea. This can be explained by the fact that supplementary foods may increase the exposure of children to different types of diseases causing pathogenic microorganisms due to contamination of food and water with different wastes [36]. Contamination of supplementary foods is very common in developing countries due to contaminated water, poor personal hygiene, inadequate cleaning of eating utensils, and inadequate storage of foods after preparation. The effect of supplementary foods is also severe if supplementary feeding is started before six months of age. This finding is in line with World Health Organization findings [44, 45]. This might be due to the fact that early complementary feeding initiation increases the risks of food borne infections, especially in regions where sanitation conditions are poor. Early intake of supplementary foods reduces the intake of breast milk and as a consequence, infants receive fewer protective factors [46–48].

This community based cross-sectional study explored that breastfeeding status at the time of the survey was statistically associated with childhood diarrheal disease. Breastfeeding has an impact on child survival of all preventive interventions. Breast milk provides all of the nutrients, vitamins and minerals an infant needs for growth. Breast milk carries antibodies that help combat disease [10, 12, 14–16, 49–52].

This study illustrated that childhood diarrheal disease was statistically associated with hygiene of the complementary foods. Diarrheal disease was highly prevalent among children who received uncooked foods and among children who didn’t eat foods immediately after cooking. Diarrhea was also very common if mothers washed their hands with water only before child meal preparation and feeding of their children. This might be due to the fact that uncooked foods, stored foods, and unhygienic hands are potential risk factors for contamination of foods and children with infectious agents. Foods that are stored under unfavorable conditions are given to infants without being heated or are inadequately reheated, resulting in an increased intake of pathogenic germs. Proper cooking and frequent hand washing with soap can reduce the load of pathogens [18, 20, 21, 23, 25].

This study revealed that childhood diarrheal disease was associated with rotavirus vaccine. Children who have been vaccinated had lower odds to develop diarrhea. This may indicate that vaccines have excellent protective efficacy against severe rotavirus gastroenteritis [18, 53–56].

### Limitation of the study

Even though, childhood diarrhea was properly defined by using the WHO diarrhea assessment tool, its occurrence was determined based on the reports of mothers, without the confirmation of physicians. Due to this phenomenon, the study might be affected by social desirability bias. However, strong efforts were made to minimize social desirability bias. However, female data collectors who were part of the community were recruited owning to their strong relationships with mothers so that could minimize the social desirability bias. The other limitation of the study was a scarcity of literature on nomads or a similar population, thus, the discussion was made on the basis of the findings of the general population.

## Conclusion

The prevalence of childhood diarrheal disease among children less than two years of age in the nomadic community of Hadaleala district was high. This high prevalence of diarrheal disease was statistically associated with the age of the children, education status of mothers, initiation of early breastfeeding, maintenance of breastfeeding, taking supplementary foods, hygiene of supplementary foods, hand washing practices of mothers, and rotavirus vaccination. This implies that a single intervention may not be sufficient to prevent childhood diarrheal disease. Therefore, the mothers should start breastfeeding early, practice exclusive breastfeeding, improve their child feeding practices and the hygiene of supplementary foods to minimize childhood diarrheal disease.

## Abbreviations

AOR: Adjusted odds ratio
CF: Complementary feeding
CI: Confidence interval
COR: Crude odds ratio
IQR: Interquartile range
Km: Kilometer
Km^2^: Kilometer square
SPSS: Statistical package for social sciences
